# The Atrioventricular Junction: A Potential Niche Region for Progenitor Cells in the Adult Human Heart

**DOI:** 10.1089/scd.2019.0075

**Published:** 2019-08-02

**Authors:** Kristina Vukusic, Mikael Sandstedt, Marianne Jonsson, Märta Jansson, Anders Oldfors, Anders Jeppsson, Göran Dellgren, Anders Lindahl, Joakim Sandstedt

**Affiliations:** ^1^ Department of Laboratory Medicine, Institute of Biomedicine, University of Gothenburg Sahlgrenska Academy, Gothenburg, Sweden.; ^2^ Department of Clinical Chemistry, Sahlgrenska University Hospital, Gothenburg, Sweden.; ^3^ Department of Pathology, Institute of Biomedicine, University of Gothenburg Sahlgrenska Academy, Gothenburg, Sweden.; ^4^ Department of Cardiothoracic Surgery, Sahlgrenska University Hospital, Gothenburg, Sweden.; ^5^ Department of Molecular and Clinical Medicine, Institute of Medicine, University of Gothenburg Sahlgrenska Academy, Gothenburg, Sweden.

**Keywords:** heart, stem cell niche, atrioventricular junction, hypoxia, cardiomyocyte proliferation

## Abstract

A stem cell niche is a microenvironment where stem cells reside in a quiescent state, until activated. In a previous rat model, we combined 5-bromo-2-deoxy-uridine labeling with activation of endogenous stem cells by physical exercise and revealed a distinct region, in the atrioventricular junction (AVj), with features of a stem cell niche. In this study, we aim to investigate whether a similar niche exists in the human heart. Paired biopsies from AVj and left ventricle (LV) were collected both from explanted hearts of organ donors, not used for transplantation (*N* = 7) and from severely failing hearts from patients undergoing heart transplantation (*N* = 7). Using antibodies, we investigated the expression of stem cell, hypoxia, proliferation and migration biomarkers. In the collagen-dense region of the AVj in donor hearts, progenitor markers, MDR1, SSEA4, ISL1, WT1, and hypoxia marker, HIF1-α, were clearly detected. The expression gradually decreased with distance from the valve. At the myocardium border in the AVj costaining of the proliferation marker Ki67 with cardiomyocyte nuclei marker PCM1 and cardiac Troponin-T (cTnT) indicated proliferation of small cardiomyocytes. In the same site we also detected ISL1^+^/WT1^+^/cTnT cells. In addition, heterogeneity in cardiomyocyte sizes was noted. Altogether, these findings indicate different developmental stages of cardiomyocytes below the region dense in stem cell marker expression. In patients suffering from heart failure the AVj region showed signs of impairment generally displaying much weaker or no expression of progenitor markers. We describe an anatomic structure in the human hearts, with features of a progenitor niche that coincided with the same region previously identified in rats with densely packed cells expressing progenitor and hypoxia markers. The data provided in this study indicate that the adult heart contains progenitor cells and that AVj might be a specific niche region from which the progenitors migrate at the time of regeneration.

## Introduction

There is a growing body of evidence supporting renewal of all cell types in the heart, including cardiomyocytes [1,2]. Several adult cardiac stem/progenitor cell populations have been described as a potential source for this regeneration, including ISL1^+^ [3], SSEA4^+^ [4,5], NKX2.5^+^ [3], WT1^+^ [6], Sca1 [7–9], MDR1^+^, and Side Population cells [10–12]. These stem cell populations could potentially be utilized in future treatment strategies for cardiovascular disease, such as heart failure.

To be able to improve the inefficient cardiac tissue regeneration, there is a need for better understanding of the stem cell organization and the specific microenvironment surrounding the stem cells. In other tissues, stem cells are known to be organized in so called stem cell niches. The niche is a reservoir of stem cells that can self-renew, divide, and give rise to new daughter cells. These daughter cells will eventually migrate out of the niche and differentiate into tissue-specific cell types needed for the normal homeostasis or as a response to injury. The concept of the stem cell niches has been established by studies of biological systems with rapid cell turnover, such as hair follicle [13,14], bone marrow, gonads, and small intestine [15,16]. There are some fundamental features that are common for stem cell niches. Generally, cadherins and catenins form intercellular junctions between the stem cells and the supportive cells. Bone marrow studies have shown that hematopoietic stem cells start to proliferate and differentiate when they lose their connection to the supportive cells [17]. Low oxygen tension (hypoxia) is another hallmark of the hematopoietic niches [18,19], where key modulators are the hypoxia-inducible factors, for example, HIF1-α. The composition of extracellular matrix (ECM) provides an optimal milieu for the maintenance of stem cells [20]. In the intervertebral disk niches it has been suggested that the stem cells migrate along the collagen fibers [21]. This migration process has been associated with the expression of Snai-1 [21].

In the niches, adult stem cells are mostly quiescent and slow cycling. Thus, DNA labeling techniques using 5-bromo-2-deoxyuridine (BrdU) have been widely used to identify the niches in different tissues [13,14]. The existence of stem cell niches in the human heart has not been shown. In mice, cardiac niches have previously been described in terms of BrdU^+^/Sca1^+^ cells between the cardiomyocytes in apex and atria [22]. Previously, we combined BrdU labeling with physical exercise to activate endogenous stem cells, and revealed a new potential niche area in the atrioventricular junction (AVj) of the adult rat heart [23]. This anatomic structure, located at the insertion points of the mitral and tricuspid valves at the ventricular myocardial border, contained slow cycling cells and showed expression of the stem cell biomarkers, MDR1 and Sca1. Therefore, in the present study, we explored the human AVj for expression of the stem cell biomarkers and well-known niche components.

There is some evidence that ischemia [24], cardiac hypertrophy, and heart failure [25] affect the cardiac stem cell populations. However, there is a lack of knowledge about the distribution of stem cells within the failing human heart and how cardiac disease affects the stem cell niches. One possible hypothesis is that during the progression of heart failure, cardiac stem cell niches become depleted. This could in itself contribute to further advancement of the heart failure. An alternative hypothesis might be that the number of stem cells increases as a response to disease, however insufficient to reverse disease itself.

We wanted to investigate whether the human adult heart contains a stem cell niche region in the AVj, similar to what we previously described in the rat heart. We also wanted to address how and if this region is affected by heart failure. For this, we obtained tissue from explanted organ donor hearts and from failing hearts of recipients of cardiac transplant.

## Materials and Methods

### Human cardiac biopsies

The study was approved by the Research Ethics Board at the Sahlgrenska Academy, University of Gothenburg, Sweden, following the Helsinki Declaration. This study was based on whole explanted hearts from which paired biopsies were obtained from two locations: the AVj, at the base of the mitral valve, and from the left ventricle (LV), both at the lateral wall.

Two groups of research subjects were included through collaborations with the Transplant Institute at the Sahlgrenska University Hospital. In the first group, cardiac tissue from multiorgan donors, 19–63 years of age, was obtained (*N* = 7). The hearts were not suitable for heart transplantation but explanted for homograft procurement and used in the present study after the valves were harvested. Organ donors with chronic heart failure were excluded. All had documentation of consent from the donor, stating that their organs can be used for other medical purposes than organ donation. The second group was patients, 39–67 years of age, with severe heart failure, undergoing cardiac transplantation surgery (*N* = 7). After signed informed consent, cardiac tissue was obtained from these failing human hearts, removed during cardiac transplantation surgery.

Medical history of the organ donors is summarized in Table 1 and transplantation patients in Table 2.

**Table 1.** Medical History of Included Multiorgan Donors

*Donor*	*Sex*	*Age*	*Cause of death*	*Other diseases*	*Exclusion criteria for transplantation*
1	F	58	Intracerebral hemorrhage	Hypertension, stroke, ischemic heart disease, atrial fibrillation, psoriasis	Ischemic heart disease
2	F	31	Benign brain tumor	None	No matching recipient
3	M	62	Subarachnoid hemorrhage	Atrial fibrillation, previous Maze surgery	MAZE surgery
4	F	44	Ischemic cerebral edema, due to suicide	Psychiatric disease, mitral valve insufficiency	Mitral valve insufficiency
5	F	50	Intracerebral hemorrhage	Previous ventricular tachycardia, suspected previous AMI, suspected Takotsubo cardiomyopathy	Suspected Takotsubo cardiomyopathy
6	F	63	Ischemic cerebral edema, due to cardiac arrest	Ischemic heart disease, hypertension, obesity, hypothyroidism, diabetes type 2, renal insufficiency, emphysema	Ischemic heart disease
7	F	19	Ischemic cerebral edema due to cardiac arrest	Anorexia	Cardiac arrest, decreased ejection fraction in the acute setting

Summarizes the clinical background of the organ donors not suitable for the cardiac transplantation.

F, female; M, male; AMI, acute myocardial infarction.

**Table 2.** Medical History of Included Cardiac Transplantation Patients

*Patient*	*Sex*	*Age*	*Cause of heart failure*	*NYHA*	*LVAD*	*Other diseases*
1	M	66	Idiopathic dilated cardiomyopathy	IIIA	Yes	PAH, stroke, and diabetes type II
2	M	67	Ischemic cardiomyopathy	IIIA	Yes	PAH, atrial fibrillation, renal insufficiency
3	M	39	Ischemic cardiomyopathy	III	No	PAH, valvular disease, diabetes mellitus type I, mild retinopathy, and nephropathy
4	M	65	Idiopathic dilated cardiomyopathy	IIIA	No	ASD, Atrial fibrillation, asthma
5	F	60	Restrictive cardiomyopathy, due to amyloidosis	IIIA	No	PAH, asthma, amyloidosis, myeloma
6	F	64	Idiopathic dilated cardiomyopathy	IIIB	No	PAH, valvular disease, atrial fibrillation, diabetes mellitus type II, hypertension, renal insufficiency, hypothyreosis, and previous ovarian cancer
7	M	50	Ischemic cardiomyopathy	IIIA	No	Stroke, diabetes mellitus type II, hypertension, immune deficiency, and previous hyperparathyroidism

Summarizes the clinical background of the included patients that underwent cardiac transplantation due to heart failure.

F, female; M, male; NYHA, New York Heart Association; LVAD, left ventricular assist device; PAH, pulmonary arterial hypertension; ASD, atrial septum deficiency.

### Histology

Biopsies from AVj and LV were collected in cold phosphate-buffered saline (PBS), embedded in Tragacanth mounting medium (Histolab Products AB, Gothenburg, Sweden), frozen in liquid nitrogen and stored at −80°C. The frozen tissue was sectioned into 7 μm serial sections. For analysis of histology, sections were stained with Hematoxylin–Eosin and Picric Sirius red.

### Immunohistochemistry

The frozen tissue sections were fixed in −20°C acetone for 10 min and washed in PBS. A blocking step followed with 2% bovine serum albumin, 0.3% Triton X-100, and 5% goat serum (Invitrogen, Carlsbad, CA), diluted in PBS for 30 min at room temperature (RT).

Primary antibodies (listed in Table 3) in different combinations were added and the sections incubated at 4°C overnight in a humidified chamber. Primary antibodies were diluted according to Table 3. Results were visualized by staining with secondary antibodies: goat anti-mouse Alexa Fluor 546, goat anti-rabbit Alexa Fluor 546, or goat anti-rabbit Alexa Fluor 647 (Invitrogen) for 1–2 h at RT. To enable triple staining, cTnT antibody was conjugated with Alexa 488 using the Zenon Kit (Invitrogen), according to the manufacturer's description using a 1:6 molar ratio. After the incubation with secondary antibody, samples were washed in PBS and incubated with a Zenon-conjugated antibody for another hour at RT. Sections were washed and fixed with Histofix (Histolab) for 15 min, washed again, and mounted with ProLong Gold Antifade Reagent with DAPI (Invitrogen).

**Table 3.** Antibodies Used for Immunohistochemistry

*Biomarker*	*Primary antibody*	*Concentration (mg/mL)*	*Dilution*	*Company*	*Catalog number*
Wt1	Rabbit monoclonal IgG	0.3	1:250	Abcam	ab89901
Islet-1	Mouse monoclonal IgG1	0.5	1:400	BD	562546
SSEA-4	Mouse monoclonal IgG3	0.5	1:50	eBioscience	14-8843-80
MDR1	Rabbit monoclonal IgG	0.3	1:250	Abcam	ab170904
cTnT	Mouse monoclonal IgG1	0.2	1:50	Thermo Fisher	MS-295-P1
PCM1	Rabbit polyclonal IgG	0.3	1:400	Atlas	HPA023374
Ki67	Mouse monoclonal IgG1	0.2	1:200	Santa Cruz	sc-2390
HIF1α	Mouse monoclonal IgG1	1.0	1:300	Novusbio	NB-100-131
CD31	Mouse monoclonal IgG1	0.5	1:100	BioLegend	303102
TE7	Mouse monoclonal IgG1	0.2	1:300	Santa Cruz	sc-73603
SNAI1	Goat polyclonal IgG	0.2	1:100	Santa Cruz	sc-10433
n-Cadherin	Mouse monoclonal IgG	0.3	1:300	BD	610920

Thermo Fischer, Thermo Fisher Scientific; Atlas, Atlas Antibodies; Santa Cruz, Santa Cruz Biotechnology.

Corresponding isotype controls for the primary antibodies were used for determining the background and did not show any specific staining.

### Image analysis

The results were visualized using an ECLIPSE Ti inverted microscope (Nikon Corporation, Tokyo, Japan) using brightfield microscopy for histology and fluorescence for immunohistochemistry (IHC) analyses. Large images were scanned automatically using a motorized board and the “stitch images” option provided in the NIS elements 4.12 software (Nikon). The size of the images was chosen not only to cover the AVj, but also parts of the myocardial wall and mitral valve, mostly 7 × 7 fields with the 20 × objective. Images were acquired at three Z-levels to capture all the nuclei in focus. For brightfield images, a Nikon DS-2Mv camera was used. Fluorescence images were acquired in a similar way with an Andor Zyla camera. Generally, four channels (DAPI, Alexa 488, Alexa 546, and Alexa 647) were acquired.

All images were exported to ImageJ software (v. 1.47h, Fiji distribution) [26] for further analysis. For each channel, displayed pixel ranges were set so that most of the background was extinguished. Isotypic controls were treated in the same way. The composite photos were used for analyses of the expression of biomarkers in large areas provided by the photos.

## Results

### Histology

**FIG. 1.**
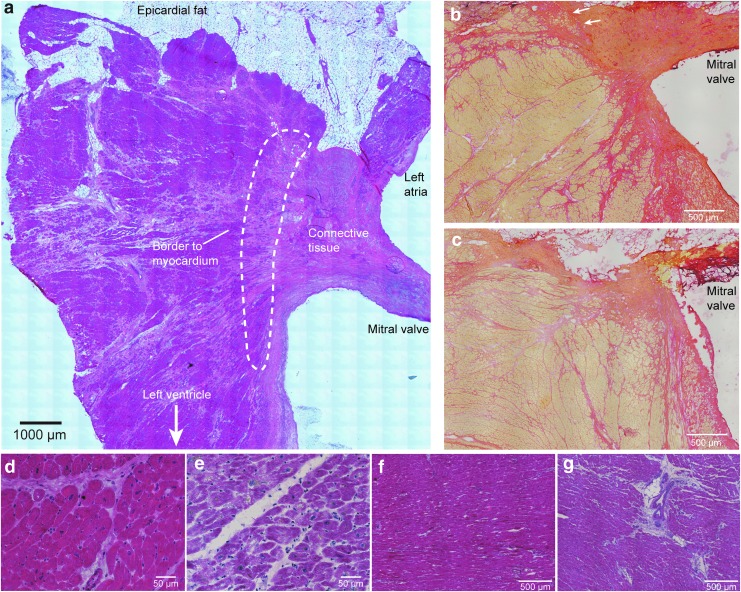
Histology and region definitions **(a)** Hematoxylin–Eosin staining of a representative AVj from an organ donor, where connective tissue and border to myocardium (within the *dashed line*) are outlined. **(b)** Picric Sirius red staining for collagen in the donor AVj. Small cardiomyocytes, at the border to myocardium, were embedded in collagen (pointed out by *arrows*). **(c)** AVj from failing hearts showed disrupted collagen fiber network and a thinner mitral valve. **(d)** Cardiomyocytes from the donor LV compared with **(e)** hypertrophic cardiomyocytes in the failing LV. **(f)** Overview of the donor LV tissue compared with **(g)** failing LV tissue. The failing heart demonstrated hypertrophy, fibrosis, and larger blood vessels. AVj, atrioventricular junction; LV, left ventricle.

Sections from AVj and LV, from both the donors and the failing explanted hearts, were stained with Hematoxylin–Eosin and Picrosirius Red staining for histology examination (Fig. 1). AVj is a region rich in ECM. Picrosirius Red staining of donor hearts showed the strongly red stained mitral valve and the radiating strands of collagen fibers continuing into the myocardium of the LV. Between the mitral valve insertion point and the border to myocardium, there is the connective tissue region. Cardiomyocytes at the myocardial border zone were small and embedded in ECM (Fig. 1b).

In contrast to the donor tissue (Fig. 1b), Picrosirius Red showed weaker staining in the AVj of the failing hearts (Fig. 1c), suggesting less collagen content. The organization of collagen fibers appeared as disrupted. The mitral valve was thinner with increased content of adipocytes at the insertion point at the ventricular side. LV tissue from failing hearts showed histopathological changes with fibrosis and hypertrophy when compared with tissue from donor hearts. Cardiomyocytes were often hollow with enlarged and irregular nuclei with lipofuscin around (Fig. 1d, e). In addition, larger blood vessels were found in LV tissue of failing hearts, and surrounded by fibrous tissue (Fig. 1f, g).

### High expression of stem cell and hypoxia markers in the donor AVj

Using different antibodies against selected biomarkers, we investigated the human cardiac tissue for the expression of stem cell, hypoxia, and migration markers, all of which have been associated with the stem cell niches. Cardiac lineage markers, cTnT for cardiomyocytes, CD31 detecting endothelial cells, and TE-7 detecting fibroblasts, were used to study colocalization. The expression of these biomarkers was compared between the AVj and the LV tissue from the same hearts.

**FIG. 2.**
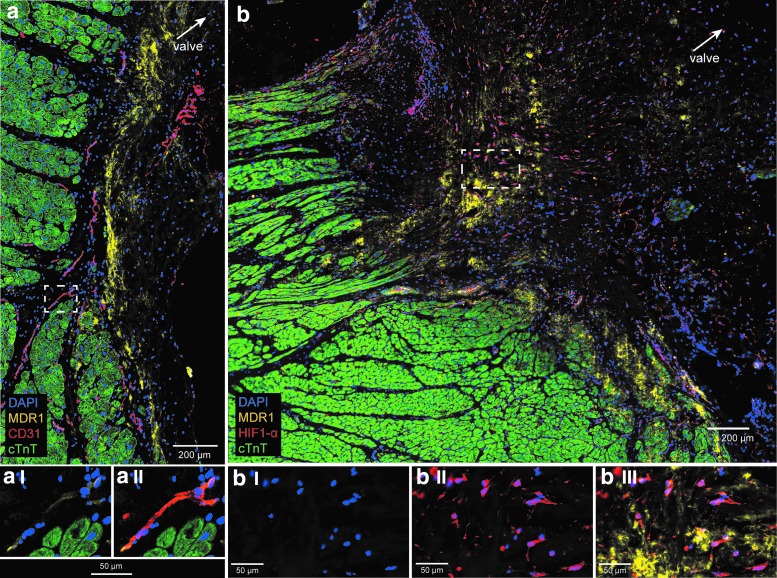
Expression of stem cell marker MDR1, endothelial marker CD31, hypoxia marker HIF1-α, and cardiomyocyte marker cTnT, in the donor AVj. The original large images were cropped to show staining in greater magnification. **(a)** A representative photo of IHC staining with antibodies against MDR1 (*yellow*), CD31 (*red*), and cTnT (*green*) showing a large streak of MDR1^+^ cells in the connective tissue region, outside of the border to myocardium, where CD31^+^ cells were absent. **(aI–II)** Further enlargement of the region within the *dashed line* in **(a)** shows clear coexpression of CD31 and MRD1 in the blood vessels. **(b)** A representative photo of IHC staining with antibodies against MDR1 (*yellow*), HIF1-α (*red*), and cTnT (*green*). HIF1-α was expressed in many nuclei in the connective tissue region. MDR1 shows a cytoplasmic/membranous expression pattern in the same region but also in the border to myocardium. **(bI–III)** Further enlargement of the region within the *dashed line* in **(b)** showing nuclear localization of HIF1-α in the connective tissue region, where high expression of MDR1 was found. cTnT, cardiac troponin T; IHC, immunohistochemistry; HIF1α, hypoxia-induced factor α; MDR1, multidrug resistance protein 1.

The connective tissue region in AVj was identified by the absence of cTnT staining that was used for orientation to the proposed niche region. In this region, MDR1 was strongly expressed in a large streak of densely packed cells. In this streak, CD31 staining was absent, indicating low vascularization (Fig. 2a). Notably, coexpression of MDR1 was also found within the CD31^+^ blood vessels nearby (Fig. 2aI, aII). In addition, hypoxia marker HIF1-α was strongly expressed in the nuclei of many cells in the connective tissue region of the AVj, colocalizing with the expression of MRD1 (Fig. 2b, bI–bIII). Dim MDR1 expression was noted in the border to myocardium and was decreasing with the distance from the valve. In the LV tissue, MRD1 and nuclear HIF1-α expression was only seen in the blood vessels (data not shown).

**FIG. 3.**
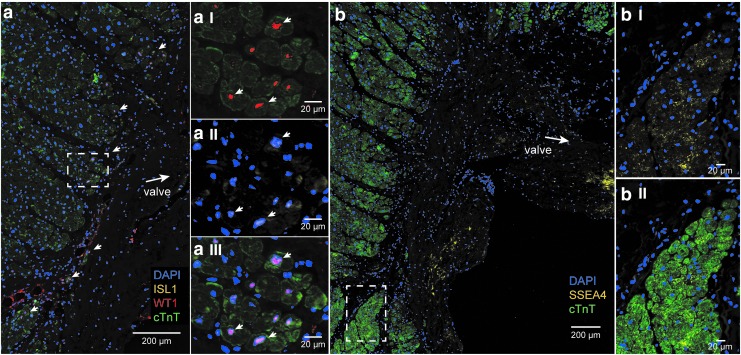
Expression of fetal cardiac stem cell markers ISL1, WT1, SSEA4, and cardiomyocyte marker cTnT, in the donor AVj **(a)** A representative photo of IHC staining with antibodies against ISl1 (*yellow*), WT1 (*red*), and cTnT (*green*) showing the clusters of WT1^+^/ISl1^+^/cTnT^+^ cells at the border to myocardium (*arrowheads*). **(aI–III)** Further enlargement of the region within the *dashed line* in **(a)** show co-expression of WT1^+^/ISl1^+^/cTnT^+^ cells (*arrows*). **(b)** A representative photo of IHC staining with antibodies against SSEA4 (*yellow*) and cTnT (*green*) showing a streak of SSEA4^+^ cells in the connective tissue region. **(bI–II)** Further enlargement of the border to myocardium shows SSEA4^+^/cTnT^+^ cells, in this region.

Furthermore, expression of the fetal cardiac stem cell markers, ISL1 and WT1, was investigated. Nuclear expression of the transcription factor ISL1 was found in the connective tissue region in the AVj. WT1 expression was absent in the connective tissue but was found in the myocardium border (Fig. 3a, aI–III). The border cardiomyocytes were small and cTnT^+^/WT1^+^/ISL1^+^. Otherwise ISL1 and WT1 did not costain. Further away from the myocardium border, WT1 was expressed by cells between cardiomyocytes and more densely at the epicardium. LV tissue from organ donors showed no expression of ISL1 and WT1 (data not shown).

Another fetal stem cell marker, the glycoprotein SSEA4^+^, showed a membranous expression pattern. SSEA4^+^ cells were found in a large streak in the connective tissue (Fig. 3b), similar to MDR1. The small border cardiomyocytes were cTnT^+^/SSEA4^+^ (Fig. 3bI–II), and the expression was decreasing with the distance from the valve. In the LV tissue, there was no SSEA4 expression (data not shown).

### Expression of proliferation and migration markers in the donor AVj

**FIG. 4.**
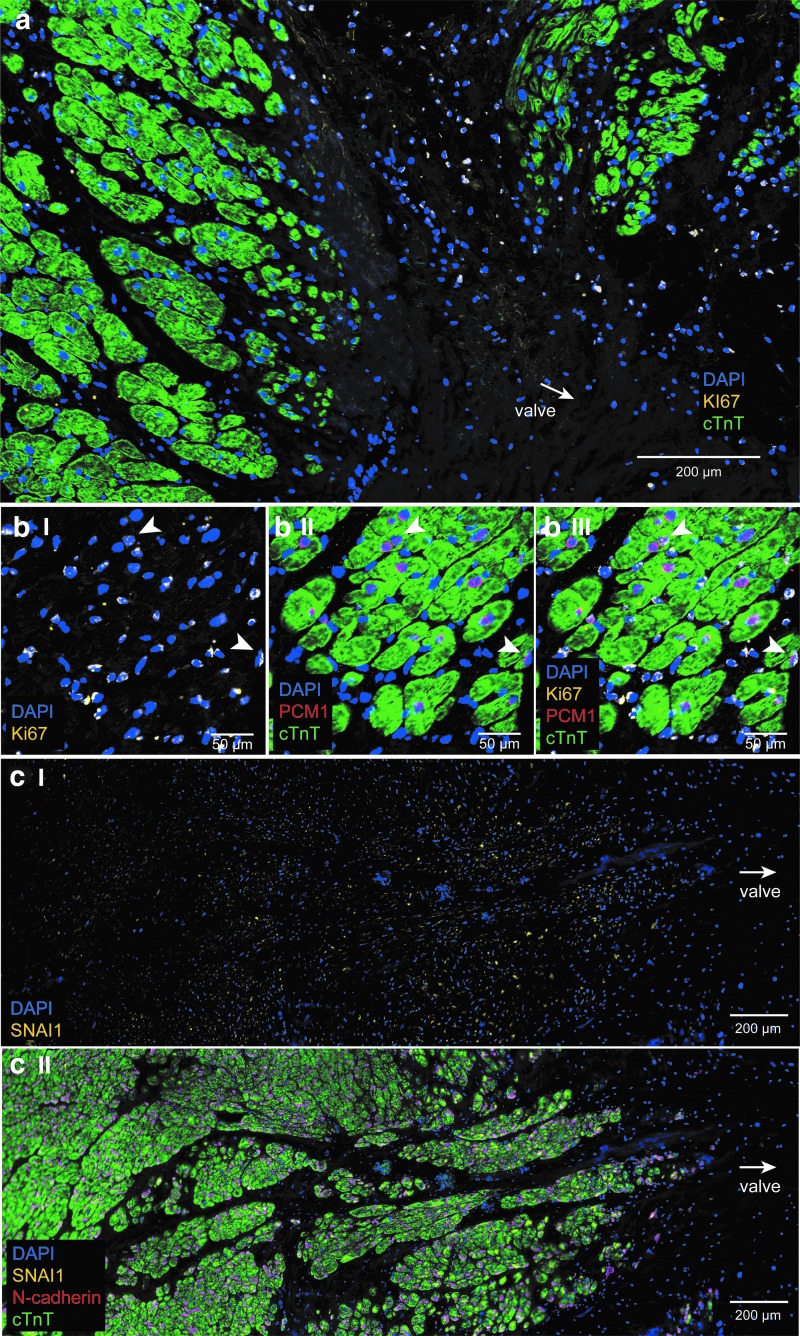
Expression of proliferation marker Ki67 (*yellow*, on the *blue* nuclei it appears as *white*), cardiomyocyte nuclei marker PCM1 (*red*), cardiomyocyte marker cTnT (*green*), migration marker SNAI1 (*yellow*), and intercellular junction protein N-cadherin (*red*), in the donor AVj. **(a)** A representative photo of IHC staining with antibodies against Ki67 and cTnT showing Ki67^+^ cells in the connective tissue region and border to myocardium. **(bI–III)** Further enlargement from border to myocardium where cTnT^+^/PCM1^+^/Ki67^+^ cells were identified (*arrowheads*), suggesting proliferating cardiomyocytes. **(cI–II)** A representative photo of IHC staining with antibodies against SNAI1, N-cadherin and cTnT showing expression of the SNAI1 and N-cadherin from the border of the myocardium.

The proliferation marker Ki67 was expressed in many cells in the connective tissue region of the the AVj in donor hearts (Fig. 4a). A marker that specifically detects human cardiomyocyte nuclei, PCM1, was studied. As expected, it was specifically expressed in the nuclei surrounded by cTnT-positive cytoplasm. Interestingly, in the border to myocardium, some PCM1^+^ nuclei were found without a cTnT^+^ cytoplasm around. Also, some Ki67^+^/PCM1^+^/cTnT^+^ cells were found here (Fig. 4bI–III). The size of the cTnT^+^ cytoplasm increased with the increasing distance from the valve. Taken together, these findings suggest different stages of cardiomyocyte development.

Migration marker Snai1 was expressed, starting from the border to myocardium, colocalizing with the cTnT (Fig. 4cI, cII). The intensity of the staining decreased with the distance from the valve and was not expressed in LV. N-cadherin stained the intercellular junctions between cardiomyocytes and the expression appeared from the border to myocardium (Fig. 4cII) and continued throughout LV. No expression was found in the connective tissue region.

### Different expression pattern of biomarkers in the failing AVj and LV

**FIG. 5.**
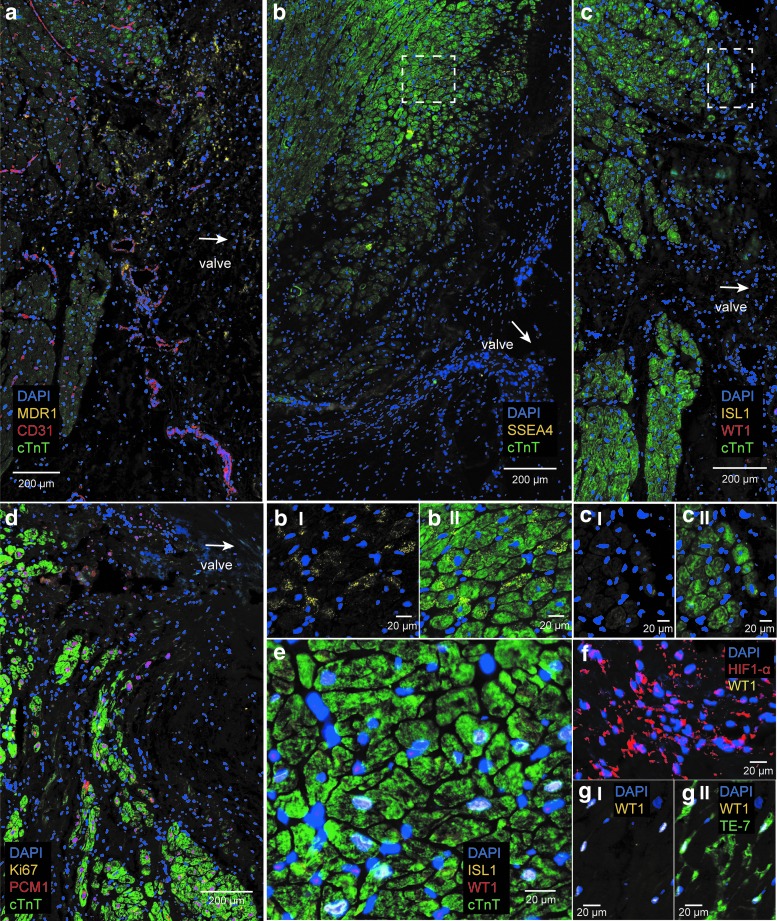
Expression of biomarkers in the failing AVj and LV. **(a)** A representative photo of IHC staining with antibodies against stem cell marker MDR1, endothelial marker CD31, and cardiomyocyte marker cTnT showing weaker expression of MDR1 in the connective tissue region compared with donor hearts. More and larger blood vessels were shown by CD31 expression. **(b)** Stem cell marker SSEA4 (*yellow*) was hardly expressed in the connective tissue region of the AVj in failing hearts. Further enlargement **(bI–II)** of the region within the *dashed line* in **(b)** shows coexpression of SSEA4 with cTnT (*green*) in the border to myocardium. **(c)** Stem cell markers ISL1 (*yellow*) and WT1 (*red*) were not expressed in the AVj of failing hearts. **(cI–II)** Further enlargement from the border to myocardium with the *dashed line* in **(c)** where no ISL1- or WT1-positive nuclei were detected. **(d)** Different stages of cardiomyocytes illustrated by cardiomyocyte nuclei marker PCM1^+^ (*red*)/cTnT-(*green*) cells at the border to myocardium and different sizes of cTnT^+^ cells. No proliferation marker Ki67^+^ (*yellow*) cells were detected. **(e)** A representative photo of IHC staining of failing LV, where stem cell markers ISL1 (*yellow*, on the *blue* nuclei it appears as *white*) and WT1 (*red*) were coexpressed by many cells. **(f)** A representative photo of hypoxia marker HIF1-α (*red*) staining showing high expression in the failing AVj. **(gI–gII)** shows coexpression of WT1 (*yellow*, on the *blue* nuclei it appears as *white*) and the fibroblast marker Te7^+^ (*green*) suggesting activation of fibroblasts in the failing LV.

In the failing hearts, a weaker expression pattern was seen for MDR1 and SSEA4 in the AVj (Fig. 5a, b). Some positive cells were found in the myocardium (Fig. 5bI–II). More and larger CD31^+^ blood vessels were observed in the AVj of failing hearts compared with the AVj of donor hearts (Fig. 5a). In the connective tissue region, a weak expression of MDR1 was noted with absence of CD31^+^ cells. Some CD31^+^ blood vessels were observed to coexpress MDR1, similar to the pattern observed in donor AVj. Hypoxia marker HIF1-α was found in nuclei of many cells in the connective tissue region in AVj of failing hearts (Fig. 5f).

In contrast to the donor hearts, no expression of the fetal cardiac stem cell markers, WT1 or ISL1, could be found in the AVj of failing hearts (Fig. 5c, cI–II).

In six of the failing hearts no expression of the proliferation marker Ki67 was found (Fig. 5d), except in adipose tissue and blood vessels. However, there was one outlier (Patient 5, Table 2) with many Ki67^+^ cells, observed in the mitral valve and the connective tissue region of AVj. Notably, PCM1^+^ nuclei without a surrounding cTnT cytoplasm were present at the border in all failing hearts (Fig. 5d).

In the LV of failing hearts, ISL1 expression was observed in large nuclei, mostly in cTnT^+^ cardiomyocytes. WT1 was expressed by cells between the cardiomyocytes (Fig. 5e). Fibroblast marker TE-7 expression colocalized frequently with WT1 expression in the failing LV (Fig. 5gI–II). WT1 was not expressed in HIF1-α^+^ cells (Fig. 5f).

Weak expression of the migration marker Snai1 was found in the border to myocardium. No expression of Snai1 was found in the LV. N-cadherin was expressed from the border to myocardium throughout the myocardium between the cardiomyocytes (data not shown).

## Discussion

Although stem cell niches have been extensively studied in other tissues, little is known regarding the heart. Therefore, even less is known about the impact of heart failure on stem cell niches. In the present study, we, for the first time, show that the AVj of the human adult heart harbors cells expressing several stem cell markers, displays different stages of cardiomyocyte development, and shows signs of hypoxia, proliferation, and migration, all considered as features of a stem cell niche. Furthermore, we observe signs of depletion of the niche region in heart failure patients.

Previously, we identified a potential stem cell niche in the AVj [23] based on BrdU label retention in a rat exercise model. In the adult human heart, however, an anatomic structure that acts as a stem cell niche has not yet been revealed. Since DNA labeling methods such as BrdU incorporation are not possible in humans, we focused on an extensive characterization of niche-associated biomarker expression by IHC in the human AVj. As comparison, tissue sections of LV were used.

During development, annulus fibrosus of AVj and valve leaflets are derived from proepicardium [27]. This dense connective tissue region forms a physical barrier between the atrial and ventricular myocardium. Histological analysis of the human donors showed high collagen content in AVj displaying a milieu rich in ECM. A specific composition of ECM is important to provide an optimal milieu for stem cell niches [20]. Heart failure and ischemia are known to induce structural changes in ECM composition such as increased mechanical stiffness and increased collagen content within the myocardium. In contrast, we noted a weaker staining of Picrosirius Red in the failing AVj and a disruption of the fiber network. This could be an indication of a dysfunctional niche structure in the failing hearts. It would therefore be of great interest to investigate the effects of heart failure on ECM composition in the AVj in more detail, to elucidate the impact of disrupted ECM on regenerative response. This was however beyond the scope of the present study.

Expression of several different cardiac stem cell markers was observed in the AVj of donor hearts, including SSEA4, MDR1, ISL1, and WT1. SSEA4 has previously been detected with IHC in human cardiac tissue [5,24] and in sheep [28]. We detected SSEA4 expression strong in the connective tissue area and costaining with cTnT^+^ small cardiomyocytes in the myocardium border of AVj. Intensity of SSEA4 staining decreased with the distance from the valve. The decrease in SSEA4 expression may be interpreted as a gradual maturation of cardiomyocytes with increased distance from the valve. Interestingly, a similar pattern was observed for BrdU staining in our previous rat label retention study [23], which further supports this hypothesis.

MDR1 was also detected in a large streak in the AVj of donor hearts, with similar expression pattern as SSEA4. In addition, MDR1 was also expressed in some blood vessel structures in both AVj and LV, as determined by MDR1/CD31 coexpression. MDR1 expression has previously been linked to side population cells in the adult mouse [29] and human [10] heart. These cells have been described as multipotent with both endothelial and cardiomyogenic differentiation capacity. It could be speculated that the observed MDR1^+^CD31^+^ cells in the AVj correspond to progenitors contributing to angiogenesis, whereas MDR1^+^CD31^−^ cells in the niche region would correspond to side population cells with a more immature phenotype.

We investigated expression of early cardiac transcription factors, ISL1 and WT1. ISL1 expression has been reported by the Grinnemo group in the outflow tract during early development, where coexpression with cTnT was reported in clusters of Isl1^+^ cells [30]. A residual expression of ISL1 could also be detected during adulthood [31]. In the present study, we found ISL1^+^/cTnT^−^ cells in the connective tissue region, but also ISL1^+^/WT1^+^/cTnT^+^ cells in the border to myocardium of donor hearts. Our findings might be a reservoir of progenitors remaining from the embryonic stage. The fetal outflow tract and the adult AVj around the mitral valve might be different nomenclature for the same anatomic region.

WT1 on the other hand is expressed by epicardial progenitors contributing to the formation of coronary vessels and interstitial fibroblasts. It has been determined that WT1 is essential for the epithelial to mesenchymal transition of these progenitor cells [32]. A continued low expression of WT1 in blood vessels of the heart during adulthood has been described [6,33]. To our knowledge, this is the first time WT1 expression has been described in a subpopulation of adult cardiomyocytes. The function of WT1 in these small cTnT^+^ cells is unclear but could together with ISL1 be interpreted as a sign of immaturity of cardiomyocytes. Further studies are needed to investigate functional relevance of WT1 as well as ISL1 in cardiomyocytes.

It has been discussed that an ultimate stem cell identifier may not exist [34]. Consequently, in the present study, we choose to analyze several different cardiac stem cell/progenitor markers. Notably, all of these were present either coexpressing or neighboring in the AVj, which supports the hypothesis that this region harbors a stem cell niche. Whether there is a hierarchy link between these different cell populations, however, remains to be determined.

Hypoxia is one of the key factors for maintenance of the quiescent state of stem cells [18,19]. Epicardium has also been identified as stem cell niches [35,36], where hypoxia was shown by capillary density quantification and HIF1-α expression [37]. In the present study, expression of the HIF1-α was found in the connective tissue region of AVj, suggesting that a similar hypoxic milieu exists in the AVj of both donor and failing hearts. Lack of CD31 expression in the streak of cells that expressed HIF1-α was noted. Interestingly, in the same region, MDR1 was highly expressed, connecting this stem cell marker to the hypoxic region.

We noted that cardiomyocyte size, based on cTnT staining, increased with the distance from the valve in the border to myocardium. Small cardiomyocytes were only present at the border. In addition, PCM1^+^ nuclei, lacking a cTnT^+^ cytoplasm, were observed just outside the myocardium in the connective tissue area. As PCM1 is specifically expressed in the nuclei of cardiomyocytes [2], this could be interpreted as an early stage in a cardiomyocyte development, derived from the nearby niche. In addition, the border to myocardium was the most active site when studying the expression of proliferation marker Ki67. A few Ki67^+^/PCM1^+^/cTnT^+^ cells were found in this area indicating proliferative capacity of subpopulation of small adult cardiomyocytes in the AVj.

Finally, effects of severe heart failure on the expression of niche-associated biomarkers in the AVj and LV were analyzed. Expression of stem cell-associated markers, SSEA4, MDR1 was either lower or absent and no expression of ISL1 or WT1 could be detected in the AVj of failing hearts. Expression of proliferation marker Ki67 was also absent in the AVj, with the exception of adipose tissue and blood vessels (data not shown). Patient 5 was noted as an outlier, with high Ki67 expression. The reason for this is unclear, but notably this patient had an extensive comorbidity that may have affected the proliferation rate. Taken together, our data indicate that the regenerative capacity of the proposed niche in AVj was impaired in the severely failing hearts.

Generally, progenitor markers were not expressed in the LV tissue in the organ donor group. In contrast, expression of ISL1 was detected in cardiomyocyte with the largest nuclei in the LV of failing heart. These ISL1^+^/cTnT^+^ large nuclei might be an effect of polyploidization. Indeed, increase in polyploidy of cardiomyocytes has previously been shown in pathological situations such as ischemic injury [38,39]. In addition, WT1 expression was detected in cells between the cardiomyocytes that also expressed fibroblast marker TE-7. This was interpreted as activation of fibroblasts as a response to stress. Reactivation of fetal gene program in biopsies from heart failure patients has been reported and appear to be enriched in the LV compared with right atrial tissue [40]. The potential of fetal genes as clinical biomarkers for heart failure pathogenesis have been discussed by several studies [41–43].

Some limitations of the present study should also be acknowledged. The medical history within both groups displayed heterogeneity. Although organ donors with chronic heart failure were excluded, some of the included donors had comorbidities that could have affected the results. The concept of the stem cell niche also includes other aspects that were not examined in the present study. Therefore, lineage-tracing studies would be required to investigate the function of the progenitors residing in the AVj.

In conclusion, we propose a potential hypoxic progenitor niche in the human AVj, which to our knowledge has not previously been described. This specific anatomic site, at the insertion point of the mitral valve, contained densely packed cells expressing early fetal cardiac markers, different stages of cardiomyocyte development, and showed signs of migration and proliferation. Based on its location, this structure may be of importance for the tissue homeostasis of the valvular apparatus as well as ventricles and atria. Our data also point toward an impairment of this niche region in patients suffering from end-stage heart failure. Identification of a potential progenitor niche in the adult human heart is an important step toward a better understanding of the basic concepts of cardiac regeneration.
